# Use of Selected Lactobacilli to Increase γ-Aminobutyric Acid (GABA) Content in Sourdough Bread Enriched with Amaranth Flour

**DOI:** 10.3390/foods8060218

**Published:** 2019-06-18

**Authors:** Manuel Venturi, Viola Galli, Niccolò Pini, Simona Guerrini, Lisa Granchi

**Affiliations:** 1 FoodMicroTeam s.r.l., Via di Santo Spirito, 14, 50125 Florence, Italy; manuel@foodmicroteam.it (M.V.); simona@foodmicroteam.it (S.G.); 2 Department of Agriculture, Food, Environment and Forestry (DAGRI), University of Florence, Piazzale delle Cascine, 18, 50144 Florence, Italy; niccolo.pini@unifi.it (N.P.); lisa.granchi@unifi.it (L.G.)

**Keywords:** sourdough, γ-aminobutyric acid GABA, amaranth flour, lactobacilli, bioactive compounds

## Abstract

γ-Aminobutyric acid (GABA) is the primary inhibitory neurotransmitter of the central nervous system and possesses various physiological functions. GABA production can be obtained thanks to lactic acid bacteria activity in different foods such as sourdoughs. Recently, breads made from blends of pseudocereals and wheat flours have attracted much attention. Amaranth is especially interesting because of its high nutritional value, having a high protein content and containing different antioxidant compounds. Therefore, this study aimed to obtain sourdough breads enriched with GABA thanks to bacterial activity and to investigate the effect of amaranth flour addition on the antioxidant and sensorial properties of bread. Eighteen lactobacilli strains were assayed for GABA production in amaranth and wheat flour liquid sourdoughs. Two strains, *Lactobacillus brevis* A7 and *Lactobacillus farciminis* A11, demonstrated high GABA producing capability; hence, they were used to prepare breads containing 20% amaranth flour. The results confirmed the capability of the two strains to increase GABA concentrations (up to 39 mg/kg) in breads. Samples with amaranth addition showed a significantly higher total phenolic content compared to the control bread (more than 15 mg GAE 100 g^−1^ dwb); sensory analysis showed that breads with amaranth were moderately acceptable. Nevertheless, their general liking evaluation was significantly lower compared to the control bread. The addition of pseudocereal to traditional wheat sourdough and selection of lactobacilli allowed the production of baked goods with enhanced GABA content and antioxidant capacity, but recipes have to be developed to increase the organoleptic acceptability of the final products.

## 1. Introduction

Sourdough fermentation represents a traditional biotechnology able to improve and enhance the overall quality of leavened bakery goods obtained using a wide variety of flours [1]. Due to the activity of the occurring microbiota (lactic acid bacteria and yeasts) and the long fermentation times, sourdough positively influences the final products, improving flavour, texture, shelf-life, and above all the nutritional and functional features of the final product [2,3,4]. Particularly, the metabolic capability of lactic acid bacteria (LAB) is fundamental to obtain these distinctive characteristics. For instance, microbial activities toward cereal proteins, such as decarboxylation, deamination, transamination, and side chain modification, may lead to the production of several compounds which have an impact both on the nutritional quality and the flavour of bakery products [5,6,7,8,9]. In this context, the production of bioactive compounds, such as γ-aminobutyric acid (GABA), by LAB during sourdough fermentation could be particularly attractive [10,11]. γ-aminobutyric acid, a non-protein aminoacid, has several physiological functions in humans: Diuretic and hypotensive activity, a tranquillizing effect, and it acts as an inhibitory neurotransmitter in sympathetic brain function [10,12,13,14,15]. In fact, foods fortified with GABA are efficient in the regulation of sleeplessness, autonomic disorders, and depression [10,16]. GABA is produced from the irreversible α-decarboxylation of l-glutamic acid, catalysed by a specific enzyme, glutamic acid decarboxylase (GAD), found in bacteria, plants and animals [5,10,17]. Some works have already shown the presence of GAD activity in LAB [18,19,20]. In this regard, fermented foods represent an excellent source of dietary GABA [5]. Furthermore, as a stable part of diet worldwide, cereal-based products enriched with GABA are of particular interest. GABA enrichment of baked goods by exploiting selected LAB, belonging to various species, has been obtained using different flours [10,11,18,19,21]. Diana et al. [11] used a strain of *Lactobacillus brevis*, isolated from cheese, for the manufacture of wheat bread with a final GABA content of 24.2 mg/100 g. Strains of *Lactococcus lactis* and *Lactobacillus plantarum* isolated from cheese were used for the production of a bread GABA enriched, using a mixture of pseudocereals and leguminous flours [18]. The selection of peculiar cereals or pseudocereals, based on their nutritional potential, may be very useful to improve the technological and functional features of baked goods [22]. Indeed, the successful replacing of standard wheat flour with unconventional flours pseudocereal or legume flours has been demonstrated [22,23]. Whole pseudocereal grains such as quinoa, amaranth, and buckwheat have abundant content of different compounds, i.e., vitamins, fatty acids, flavonoids and phenolic acids, with a known positive impact on human health [24,25,26,27]. For these reasons, an increment of these cereals’ consumption in the daily human diet has been recommended [24,28,29,30]. Among the pseudocereal group, amaranth is of particular interest because of its high nutritional value, having a high protein content, a high concentration of essential aminoacids [31], and containing different antioxidant compounds. Hence, the potential of sourdough fermentation, combined with amaranth supplement, could be a tool to develop new healthy baked products. Nevertheless, the sensory quality of the final products has to be taken into account since it is strongly influenced by the addition of amaranth flour [32]. Therefore, this study aimed to screen GABA-producing lactobacilli in order to obtain naturally GABA-enriched sourdough breads, and concurrently to investigate the effect of 20% addition of amaranth flour on the antioxidant properties of the final products. The sensory quality of the breads was also evaluated to determine the contribution of lactobacilli fermentation and amaranth flour addition on the organoleptic characteristics.

## 2. Materials and Methods 

### 2.1. Lactobacilli Strains and Culture Conditions

Eighteen lactobacilli strains of five species (*L. brevis*, *L. farciminis*, *L. plantarum*, *L. rossiae* and *L. sanfranciscensis*), previously isolated from six Italian sourdoughs and belonging to the culture collection of the Department of Agriculture, Food, Environment and Forestry (DAGRI) of the University of Florence, were used in this study (Table 1).

### 2.2. Liquid Sourdough Fermentations

The lactobacilli strains, grown overnight in MR3i broth [33], were singly inoculated (9.0 log CFU/mL) into wheat and amaranth flour doughs. The sourdoughs were prepared by mixing water and flour to obtain a dough yield (DY), i.e., the percent ratio of the weight of the dough to the weight of the flour, of 333 and 667, for wheat and amaranth liquid sourdoughs, respectively. The samples were incubated for 6 h at 30 °C under stirring conditions (ca. 100 rpm). Control and acid control doughs (acidified to pH 3.5 by lactic acid addition) without bacterial inoculum were prepared and incubated under the same conditions. At the end of the fermentation time, sourdough samples were taken in order to perform analysis of GABA content and select the highest-GABA-producing lactobacilli strains.

### 2.3. Sourdough Fermentation and Bread Making

Six breads were prepared according to the recipes reported in Table 2. For each bread, a prefermented dough was prepared. Four doughs were inoculated with only baker’s yeast (termed as PFC W, PFAC W, PFC Am and PFAC Am) and two with the inoculum of baker’s yeast and the selected lactobacilli strains: *Lactobacillus brevis* A7 and *Lactobacillus farciminis* A11 (termed PFSD A7 and PFSD A11, respectively) (Table 2).

Amaranth flour was added as 20% of the total flour in four doughs; acid control doughs were acidified by lactic acid to 3.5. The prefermented doughs were fermented for 18 h at 30 °C and then added to the final mixtures. In the final dough (Table 2) the ingredients were added at the same time and mixed for 10 min in a twin arms mixer (model RS12, Bernardi, Italy). The doughs were placed in the trays at 30 °C with 88–90% relative humidity for 3 h. Samples were taken at the beginning and at the end of the leavening time. Finally, doughs were baked at 180 °C for 15 min.

### 2.4. Monitoring of Selected Lactobacilli Strains in Sourdough Fermentations

A Randomly Amplified Polymorphic DNA (RAPD) analysis was performed according to Venturi et al. [34] to assess the occurrence of the selected lactobacilli in liquid and firm sourdoughs and final doughs. DNA was amplified using the following primers: OPL-05 (50 ACGCAGGCA 30), designed by Seseña et al. [35], and MV1 (50 GGACGCTTCTG 30) designed by Venturi et al. [34]. The random primer MV1 was used separately, while OPL-05 was used along with the primer RD1 (5’ GCTTAAGGAGGTGATCCAGCC 3’). DNA amplification was performed as described by Reguant and Bordons [36]. Amplification products were separated (at 100 V for 2.5 h) on 1.4% (*w*/*v*) agarose gel (Lonza Group Ltd, Basel, Switzerland); containing ethidium bromide (Sigma e Aldrich, St Louis, MI, USA) and TEB buffer (1 M Tris, 10 mM EDTA, 0.9 M boric acid, pH 8.3). The resulting profiles were captured as images after UV transillumination, and compared to those previously obtained for each bacterial strain. 

### 2.5. Determination of pH, Total Titratable Acidity, Volume Increase, and Enumeration of Cultivable Bacteria and Yeasts

Ten grams of dough sample were transferred into 90 mL of sterile physiological solution, homogenized for 2 min in a Stomacher Lab Blender 400 (Seward Ltd, Worthing, West Sussex, UK). After decimal dilutions, 100 µL of these suspensions were plated for cell enumeration using MR3i medium for the lactobacilli and MYPG for the baker’s yeast using the pour plate method. Lactobacilli were counted after incubation for 48–72 h at 30 °C under anaerobic conditions. Yeasts, plated on MYPG agar containing sodium propionate (2 g/L), were counted after incubation for 48 h at 30 °C under aerobic conditions. Plate counts were performed in duplicate. The pH values were determined by a pH-meter (Metrohm Italiana Srl, Varese, Italy). Total titratable acidity (TTA) was measured on 10 g of dough samples, which were homogenized with 90 mL of distilled water for 3 min and expressed as the amount (mL) of 0.1 N NaOH to achieve a pH of 8.5. To assess sourdough increase of volume, 100 g of each dough were placed in a graduated cylinder (0.5 L). The volume of the doughs (in mL) was recorded immediately (t_0_) and after 3 h of fermentation at 30 °C. The leavening was calculated using the following formula: ((V_3_ − V_0_)/V_0_) × 100, where V_3_ was the volume after the 3 h fermentation and V_0_ was the initial volume.

### 2.6. Determination of Lactic Acid by HPLC

Bread samples were diluted ten times with distilled water and then filtered by Amicon^®^ Ultra-4 Centrifugal Filters (3000 Da NMWL) (Merck Millipore) before the injection for lactic acid determination by high-performance liquid chromatography (HPLC) analysis (Varian Inc., Palo Alto, CA, USA) connected to a refractive index detector (Knauer K-2301, Knauer GmbH, Berlin, Germany) and UV detector (λ = 210). Elution was performed at 65 °C with 0.01 N H_2_SO_4_ eluent at a flow rate of 0.6 mL/min. Data were collected and analysed by using the Galaxie software (Varian Inc., Palo Alto, CA, USA). Quantitative analysis was carried out by a standard curve.

### 2.7. Total Phenols Assay by Folin–Ciocalteau Reagent

Total phenols content of bread samples was determined according to Alvarez-Jubete et al. [37], with some modifications. Bread samples were dried for 24 h at 60 °C and ground. 1.25 g were weighted and added to 25 mL methanol. Samples were vortexed and left in horizontal shaking for 24 h, then centrifuged for 10 min at 2000× *g*. The final extracts were obtained by filtering 10 mL of the supernatant through 0.22 μm PTFE syringe filters (Whatman) and they were stored at −20 °C until analysis. The reaction mixture consisted of 100 μL of methanolic bread extract, 100 μL of methanol, 100 μL of Folin–Ciocalteu reagent and 700 μL of Na_2_CO_3_. The samples were vortexed immediately, and the tubes were incubated in the dark for 20 min at room temperature. After incubation, all samples were centrifuged at 11,300× *g* for 3 min. The absorbance of the supernatant was then measured at 735 nm in 1 mL plastic cuvette using a spectrophotometer (Cary 50 Scan, Varian Inc., Palo Alto, CA, USA). Gallic acid was used as a standard and a calibration curve was prepared with a range of concentrations from 10−200 mg/L. The results are expressed in mg of gallic acid equivalent per 100 g of dry-weight basis (mg GAE 100 g^−1^ dwb).

### 2.8. Antioxidant Capacity by DPPH Assay 

The free radical scavenging capacity of methanolic bread extracts was determined using the stable 2,2-diphenyl-1-picrylhydrazyl radical (DPPH). The scavenging effect was measured according to the method of Alvarez-Jubete et al. [37]. Also, 500 µL of extracts were added to 500 µL of DPPH methanolic solution (0.05 mg/mL). After vortexing, the mixture was left for 40 min at room temperature, and the absorbance of the resulting solution was read at 517 nm. The absorbance measured after 40 min was used for the calculation of the antioxidant capacity according to the following formula: DPPH radical-scavenging activity (%): ((blank absorbance − sample absorbance)/blank absorbance) × 100. Butylated hydroxytoluene (BHT) was also assayed as antioxidant references.

### 2.9. HPLC Determination of GABA Content 

GABA content was determined on the water-soluble extracts of liquid doughs and breads. The extracts were obtained by extracting the samples with sterile distilled water (1:3 *w*/*v*), held at 4 °C for 1 h and vortexed at 15-min intervals. Samples were finally centrifuged at 14,000× *g* for 20 min. The supernatants, containing the water-soluble fraction, were used for GABA quantification. Before the injection, the samples were prepared according to Tuberoso et al. [38]. The reaction mixture consisted of 100 µL of sample extracts, 5 µL of 100 mg/L eptilammine (internal standard, IS), 200 µL of dansyl chloride solution (derivatization agent) and 0.2 M Na_2_B_4_O_7_·10H_2_O (pH 9.3) solution up to a final volume of 1000 µL. The mixture was incubated for 30 min at 40 °C in a Termoblok and centrifuged at 11,300× *g* for 10 min. The supernatant was recovered and diluted with MeOH (1:1 *v*/*v*) for HPLC analysis. Separation was obtained with a Phenomenex Gemini C18 110A column (150 4.60 mm, 3 lm; Chemtek Analitica, Anzola Emilia, Bologna, Italy) connected to fluorimetric detector (Jasco Europe, Cremella, LC, Italy) with wavelengths set at 293 nm (Ex) and 492 nm (Em) under the following conditions: Mobile phases buffer acetate/CH_3_CN (pH 4.1) and acetonitrile, flow rate 0.8 mL/min, column temperature 25 °C. The quantitative analysis was performed using calibration graphs constructed according to the internal standard method.

### 2.10. Sensory Evaluation

Sensory evaluation of lactobacilli inoculated breads and non-acidified control breads was carried out by 46 panellists (15 male and 31 female) aged 21–65 years old. Breads were cut into pieces of 3 cm × 3 cm and randomly codified before serving. Colour, aroma, consistency, and general liking were evaluated using the hedonic 9-point scale (from “1-dislike extremely” to “9-like extremely”) [39]. Panellists have also optionally chosen among 13 attributes for the taste of breads: Salty, vapid, sweet, earthy, strange taste, sour, delicate, tasty, astringent, not to eat, gummy, interesting, and persistent [24].

### 2.11. Statistical Analysis

Chemical and microbiological determinations, performed in duplicate, were elaborated according to *t*-Test procedures or nonparametric one-way ANOVA followed by Tukey’s Test (Statistica 7.0 software package, Stat Software Inc., Tulsa, OK, USA). Differences were reported at a significance level of *p* ≤ 0.05.

## 3. Results and Discussion

### 3.1. Selection of GABA-Producing Lactobacilli Strains

Eighteen lactobacilli strains were singly inoculated in liquid doughs made with either wheat or amaranth flour and incubated at 30 °C for 6 h in order to evaluate the GABA production. All the strains were able to acidify the doughs although some differences were registered. The final pH values ranged from ca 3.50−4.10 with an average decrease of 2.35 ± 0.13 in wheat sourdoughs. Due to the lower flour content (DY = 667) and a different buffering capacity, the final pH of amaranth sourdoughs resulted higher, in a range from 4.10−5.10, with an average pH decrease of 1.74 ± 0.41. The most acidifying strains, both in wheat and amaranth flour, were *L. brevis* A7, *L. farciminis* A11, *L. farciminis* B7 and *L. rossiae* Ga12. The values of pH of the non-inoculated doughs (C) were 5.96 ± 0.05 and 6.20 ± 0.11 for wheat and amaranth dough, respectively. After 6 h of fermentation GABA concentrations were determined.

**Figure 1:**
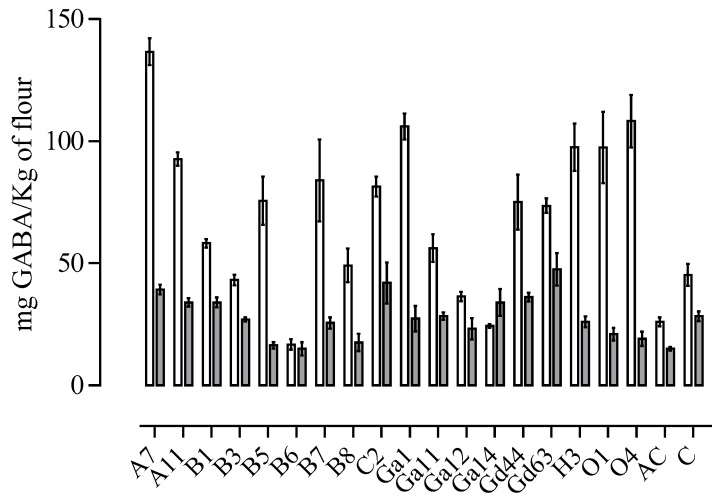
Production of GABA after 6 h fermentation in wheat (white bar) and amaranth (grey bar) sourdoughs obtained with 18 lactobacilli strains singly inoculated (data are expressed as mean ± coefficient of variation %). C—control; AC—acid control.

Results showed a variability (*p* ≤ 0.05) among the tested strains in both the flours, even if GABA concentrations were generally higher in wheat sourdoughs (Figure 1). Differences in GABA concentration in sourdoughs produced by the two flours could be due to higher glutamic acid content in wheat flour, since it is the substrate for glutamic acid decarboxylase enzyme (GAD). In agreement with the literature, the GABA producing activity was not related to the species, resulting in strain dependency [40]. In wheat sourdoughs, six lactobacilli strains displayed GABA concentrations significantly higher than the control and the acidified control, whereas in amaranth sourdoughs this trend was observed only for one strain (*L. rossiae* Gd63). Particularly, the highest (*p* ≤ 0.05) GABA concentration in wheat sourdoughs was found in the dough inoculated with *L. brevis* A7 (136.62 ± 4.00 mg/kg of flour), followed by *L. plantarum* O4, *L*. *brevis* Ga1, *L. farciminis* H3, *L. rossiae* O1, *L. farciminis* A11. The strain *L. rossiae* Gd63 displayed the highest GABA production in amaranth sourdough fermentation (47.6 ± 14.3 mg/kg of flour), although other six lactobacilli strains (*L. plantarum* C2, *L. brevis* A7, *L. sanfranciscensis* Gd44, *L. brevis* B1, *L. rossiae* Ga14 and *L. farciminis* A11) did not show statistical differences from the GABA amounts produced by the best performing strain. Hence, based on the reported results concerning GABA production and acidification ability, *L. farciminis* A11 and *L. brevis* A7 were selected to prepare firm sourdoughs and breads.

### 3.2. Sourdough Bread Fermentation with Selected Lactobacilli Strains

Breads were prepared with the addition of 20% of amaranth flour. This percentage was chosen because it is considered appropriate by various authors in order to maintain product structural quality [41], preserve the principal nutritional benefit of this ingredient [42], and meet sensory approval [43]. Table 3 shows the main technological and microbiological characteristics at the end of fermentation of the sourdoughs prepared with the inoculum of *L. brevis* A7 (SD-A7) and *L. farciminis* A11 (SD-A11). The occurrence of the two inoculated strains throughout the process was confirmed by molecular analysis. In particular, all the lactobacilli isolates from sourdoughs displayed solely the genotypic patterns of *L. brevis* A7 or *L. farciminis* A11.

The 20% amaranth incorporation did not affect the considered features. At the end of leavening time, both the sourdough pH decreased of about 0.88, reaching a value of 4.35 ± 0.03 for SD-A7 and 4.60 ± 0.18 for SD-A11, statistically different from each other and showing proper acidification [44,45]. SD-A11 final total titratable acidity was lower compared to SD-A7, while the TTA increase did not point out any significant differences. Regarding organic acids production, the highest content of lactic acid was found in SD-A7 dough (4.60 ± 0.63 g/kg). Both the samples doubled their volume, indicating an adequate leavening process. Microbiological analyses did not show any significant differences in the microorganism concentrations at the end of the fermentation. *L. farciminis* A11 and *L. brevis* A7 reached a concentration of about 8.55 log CFU/g, which is a typical value in sourdough [46], and *S. cerevisiae* reached a concentration of 7.37 log CFU/g.

### 3.3. Phenolic Content and Antioxidant Capacity of Breads

**Figure 2:**
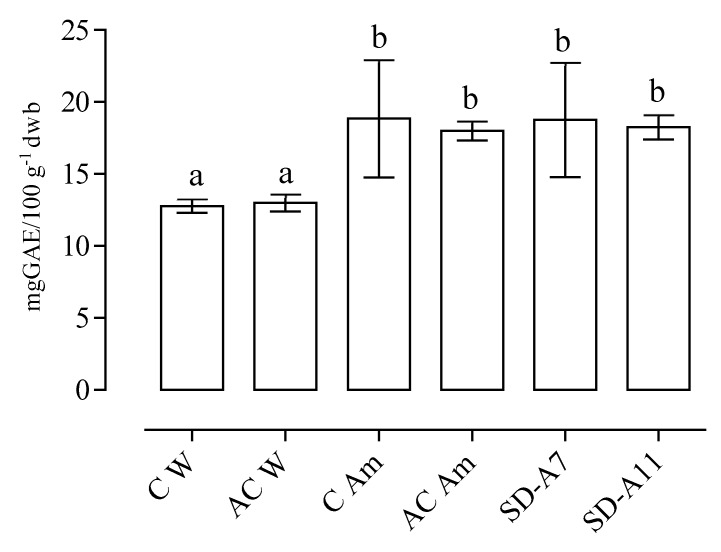
Total phenolic content (expressed as mg of gallic acid equivalents GAE100 g^−1^ dwb) of the breads (data are expressed as mean ± coefficient of variation %). C W—wheat control bread; AC W—wheat acid control bread; C Am—amaranth control bread; AC Am—amaranth acid control bread; SD-A7—*L. brevis* A7 bread; SD-A11—*L. farciminis* A11 bread. Different letters (**a**,**b**) indicate significant differences (*p* ≤ 0.05).

The results of the total phenolic content, determined on the methanolic extracts of all the breads, are shown in Figure 2.

Results indicated that amaranth flour addition significantly increased the phenolic content of breads. Indeed, whole pseudocereal grains are rich in this class of minor components, therefore their use as a supplement can increase the nutritional value of baked goods. Data showed that samples integrated with 20% of amaranth flour displayed a phenolic content of more than 15 mg GAE 100 g^−1^ dwb, which was significantly higher than the control breads, C W and AC W, made only with wheat flour (*p* ≤ 0.05). These results are in agreement with those reported by Chlopicka et al. [24], which showed a higher content of phenolic compounds in breads enriched with amaranth flour, compared to a control bread without this pseudocereal addition.

**Figure 3:**
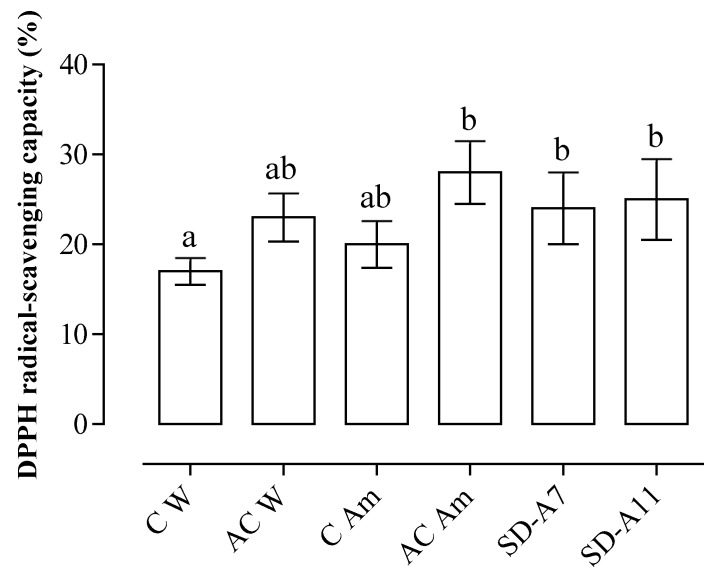
Radical-scavenging capacity (%) of the breads (data are expressed as mean ± coefficient of variation %). C W—wheat control bread; AC W—wheat acid control bread; C Am—amaranth control bread; AC Am—amaranth acid control bread; SD-A7—*L. brevis* A7 bread; SD-A11—*L. farciminis* A11 bread. Different letters (**a**,**b**) indicate significant differences (*p* ≤ 0.05).

The radical-scavenging capacity of the breads was tested by the DPPH method (Figure 3).

The antioxidant capacity of control bread made with only wheat flour result was the lowest among the samples (less than 17%), while AC Am, SD-A7 and SD-A11 showed the highest values (*p* ≤ 0.05), up to 28%. According to Vollmannova et al. [47], total phenolic content was partially correlated to the antioxidant capacity; indeed, AC Am, SD-A7 and SD-A11 showed the highest values for both these parameters. Amaranth addition only slightly increased the antioxidant capacity, on the contrary, the acidification seems to have a higher role in increasing this activity. In fact, both the chemically acidified controls showed higher values than the controls. Hence, the combination of amaranth addition and acidification might be responsible of the observed phenomenon. In this context, it was demonstrated that acidification, as a consequence of microbial fermentation, can increase the levels of easily-extractable phenolic compounds and their bioavailability [4,48,49].

### 3.4. GABA Content of Breads

**Figure 4:**
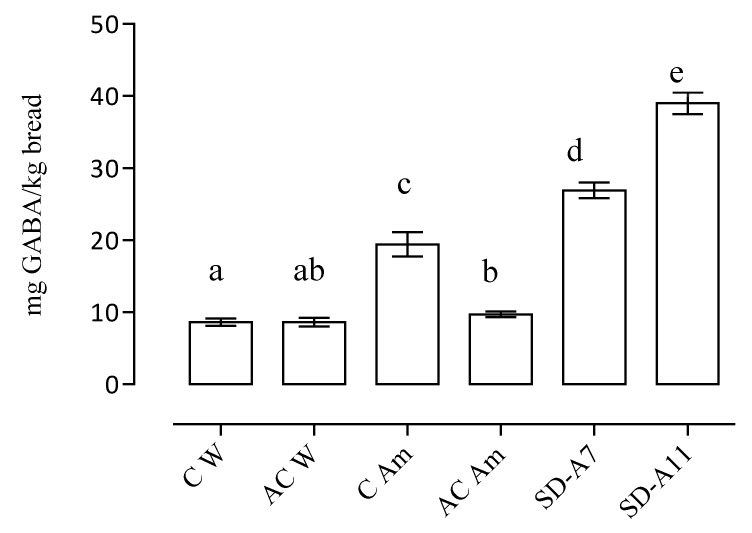
GABA concentration (mg/kg) in the breads (data are expressed as mean ± standard deviation). C W—wheat control bread; AC W—wheat acid control bread; C Am—amaranth control bread; AC Am—amaranth acid control bread; SD-A7—*L. brevis* A7 bread; SD-A11—*L. farciminis* A11 bread. Different letters (**a**–**c**) indicate significant differences (*p* ≤ 0.05).

GABA concentrations in the breads prepared by lactobacilli inoculated sourdoughs and in the control breads are reported in Figure 4.

GABA content in the breads ranged from 8.0–39.0 mg/kg. Sourdough breads showed the highest concentration with values of 26.9 ± 1.53 and 39.0 ± 1.53 mg/kg for SD-A7 and SD-A11, respectively, confirming the capability of LAB to produce GABA. The lowest content was detected in the control breads with 100% of wheat flour, less than 10 mg/kg. Compared to the control wheat breads, the GABA increase of SD-A11 was of about 350%. Other studies exploited high GABA producing biotype of *Lactobacillus plantarum* and *Lactococcus lactis* in order to obtain a bread with improved nutritional features [10,18,19]. The ability of *L. brevis* strains to synthetize GABA was reported in various food matrices such as cheese [40], yoghurt [50], black raspberry juice [51], and wheat sourdough [11], while this capability was observed only in a strain of *L. farciminis* isolated from Myanmar traditional fermented fishery products with boiled rice [52]. To the best of our knowledge, this was the first time in which GABA production by a strains of *L. farciminis* was exploited in a bakery product.

### 3.5. Sensory Evaluation

To evaluate the contribution of amaranth flour and lactobacilli fermentation on organoleptic characteristics of breads, a sensory evaluation was carried out with a panel of 46 members. Table 4 shows the results of a 9-point hedonic scale analysis of colour, odour, consistency, and general liking.

**Figure 5:**
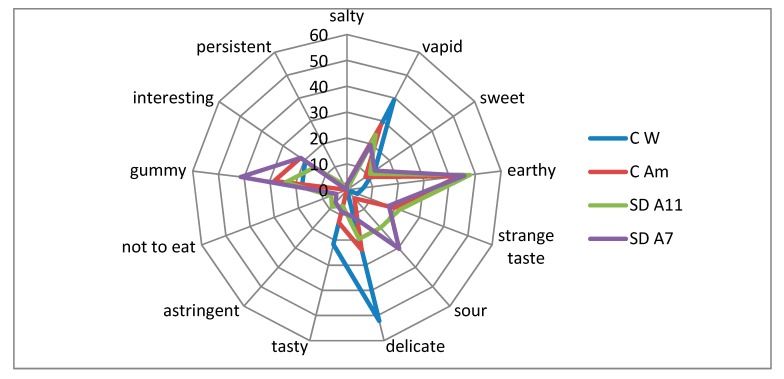
Spider plot of the sensory evaluation (%) of breads. Blue line: % of positive answers given to wheat control bread (C W); red line: % of positive answers given to amaranth control bread (C Am); green line: % of positive answers given to *L. farciminis* A11 sourdough bread (SD-A11); purple line: % of positive answers given to *L. brevis* A7 sourdough bread (SD-A7).

Values of colour and aroma were not significantly different among the breads, ranging from 6–6.7 and from 5.3–6.1, hence indicating a moderate appreciation. On the contrary, the consistency of bread was negatively affected by the addition of amaranth flour; in fact, the best value was registered for the control wheat bread (6.3 ± 1.4), statistically higher (*p* ≤ 0.05) compared to the inoculated amaranth breads (5.1–5.2). General liking of the 100% wheat control bread showed a significantly higher value (6.3 ± 1.5) compared to bread with amaranth replacement; however, none of the breads were negatively evaluated by the panellists. These results pointed out that substituting 20% of wheat flour with amaranth flour in bread was not advantageous for bread sensory evaluation, even if the panellists gave a moderate appreciation. In addition, other authors [42] reported that, considering the better nutritional features, consumers would choose to consume amaranth bread instead of common wheat bread, even if the taste is different. Furthermore, the spider plot in Figure 5 shows the different attributes chosen by panellists to describe breads.

Amaranth clearly characterized bread for earthy taste (about 50% panellists for all the amaranth breads). More than 50% panellists defined the wheat control bread as delicate, even if 40% also defined breads as vapid with respect to 20–30% of the breads with amaranth. In particular, panellists declared that SD-A7 bread was sour (30%) and gummy (more than 40%). Nevertheless, about 20% of panellists described as interesting the taste of all the breads, and only a few testers (about 5%) declared SD amaranth breads “not to eat”, in contrast with the 10–30% reported in other experiments [24]. Sensory analysis highlighted some differences between the two sourdough amaranth breads. SD-A7 bread taste was described as less delicate, gummier, and sourer, but also more interesting compared to SD-A11 bread. However, results showed that the 20% amaranth incorporation did not strongly reduce the breads’ appreciation.

## 4. Conclusions

The screening of GABA-producing lactobacilli in liquid sourdoughs led to the selection of two strains belonging to *L. farciminis* and *L. brevis* species. These strains were able to increase GABA concentration (up to 350%) in breads enriched with 20% of amaranth flour, with a final content of 26.9 ± 1.53 and 39.0 ± 1.53 mg/kg respectively. The final products showed higher antioxidant activity and an increased content of total phenolic compounds compared to the wheat control bread. Sensory evaluation indicated moderate acceptability of breads with amaranth flour, mainly characterized by an earthy taste. Although organoleptic acceptability of the final products could be improved, the combination of selected lactobacilli and pseudocereal flours can be a suitable tool for the production of innovative baked goods with improved nutritional features.

## Figures and Tables

**Table 1:** Lactobacilli strains and the isolation source.

Lactobacilli Strains	Italian Sourdough
*L. brevis* A7; *L. farciminis* A11	*Schiacciata* (flat, salty bread)
*L. brevis* B1; *L. sanfranciscensis* B3; *L. farciminis* B5; *L. farciminis* B7; *L. rossiae* B6; *L. farciminis* B8	Bread
*L. plantarum* C2	Tuscan bread
*L. brevis* Ga1; *L. rossiae* Ga11; *L. rossiae* Ga12; *L. rossiae* Ga14; *L. sanfranciscensis* Gd44; *L. rossiae* Gd63	*Lagaccio* biscuit
*L. farciminis* H3	Ancient grain bread
*L. rossiae* O1; *L. plantarum* O4	*Panettone* cake

**Table 2:** Recipes for pre-fermented dough manufacture and bread making and related dough yield (dough weight × 100/flour weight). PF—prefermented; C—control; AC—acid control; SD—sourdough; W—wheat flour; Am—Amaranth flour; A7—*L. brevis* A7 strain; A11—*L. farciminis* A11 strain.

**Prefermented Dough**						
**Ingredients (% on Total Flour)**	**PFC W**	**PFAC W**	**PFC Am**	**PFAC Am**	**PFSD A7**	**PFSD A11**
Wheat flour	100	100	80	80	80	80
Amaranth flour	--	--	20	20	20	20
Water	58	58	58	58	58	58
Baker’s yeast	1	1	1	1	1	1
*L. brevis* A7 (log (CFU/g))	--	--	--	--	9	--
*L. farciminis* A11 (log (CFU/g))	--	--	--	--	--	9
**Bread**						
**Ingredients (% on total flour)**	**C W**	**AC W**	**C Am**	**AC Am**	**SD-A7**	**SD-A11**
Prefermented dough	50	50	50	50	50	50
Wheat flour	100	100	80	80	80	80
Amaranth flour	--	--	20	20	20	20
Water	58	58	58	58	58	58
Dough yield	158	158	158	158	158	158

**Table 3:** Values (mean ± standard deviation) of pH, Total Titratable Acidity (TTA; mL NaOH), lactic acid (g/kg), volume increase (%), and microorganism (lactobacilli and baker’s yeast) concentrations (log (CFU/g)) after sourdough fermentations carried out by *L. brevis* A7 (SD-A7) and *L. farciminis* A11 (SD-A11).

Dough	Final pH	ΔpH	Final TTA	ΔTTA	Lactic acid	ΔV/V0 × 100	Lactobacilli	Yeasts
SD-A7	4.35 ± 0.03 ^a^	0.95 ± 0.39 ^a^	6.90 ± 0.14 ^b^	2.80 ± 0.66 ^a^	4.60 ± 0.63 ^b^	105 ± 7.07 ^a^	8.50 ± 0.72 ^a^	7.35 ± 0.64 ^a^
SD-A11	4.60 ± 0.18 ^b^	0.82 ± 0.10 ^a^	6.45 ± 0.07 ^a^	2.95 ± 0.78 ^a^	3.20 ± 0.56 ^a^	105 ± 21.2 ^a^	8.59 ± 0.73 ^a^	7.40 ± 0.62 ^a^

ΔV = difference between the final and the initial value; V_0_—initial volume. Values in the same column with different letters (**a**,**b**) are significantly different (*p* ≤ 0.05).

**Table 4:** Sensory evaluation of breads (mean ± standard deviation). Values were determined with a 9-point scale (extremely disliked = 1, neither like, nor dislike = 5, extremely liked = 9). C W—wheat control bread; C Am—amaranth control bread; SD-A7—*L. brevis* A7 bread; SD-A11—*L. farciminis* A11 bread.

	C W	C Am	SD-A7	SD-A11
Colour	6.7 ± 1.3 ^a^	6.2 ± 1.2 ^a^	6.1 ± 1.2 ^a^	6.0 ± 1.5 ^a^
Aroma	6.1 ± 1.5 ^a^	5.6 ± 1.6 ^a^	5.6 ± 1.5 ^a^	5.3 ± 2.0 ^a^
Consistency	6.3 ± 1.4 ^b^	5.8 ± 1.6 ^ab^	5.1 ± 1.6 ^a^	5.2 ± 1.5 ^a^
General liking	6.3 ± 1.5 ^b^	5.3 ± 1.6 ^a^	4.9 ± 1.6 ^a^	4.9 ± 1.8 ^a^

Values in the same column with different letters (**a**,**b**) are significantly different (*p* ≤ 0.05).
